# Changes in dietary habits following a colon cancer diagnosis: insights from the prospective ECHO (Eating habits CHanges in Oncologic patients) STUDY 2.0

**DOI:** 10.3389/fonc.2026.1843736

**Published:** 2026-05-21

**Authors:** Greta Caprara, Ilaria Busonera, Cristina Mazzi, Fabiana Marchetti, Daniela Bencardino, Marianna Ferrari, Maria Tieri, Lucilla Titta, Giada Magionesi, Margherita Marchiotto, Matteo Valerio, Matteo Verzè, Stefania Gori

**Affiliations:** 1 Department of Experimental Oncology, IEO, European Institute of Oncology, IRCCS, Milano, Italy; 2 Medical Oncology Unit, IRCCS Sacro Cuore Don Calabria Hospital, Negrar di Valpolicella, Verona, Italy; 3 Biostatistics Service, IRCCS Sacro Cuore Don Calabria Hospital, Negrar di Valpolicella, Verona, Italy; 4 Data Management Service, IRCCS Sacro Cuore Don Calabria Hospital, Negrar di Valpolicella, Verona, Italy; 5 IRCCS Sacro Cuore Don Calabria Hospital, Negrar Di Valpolicella, Verona, Italy

**Keywords:** colorectal cancer, dietary changes, health information, healthy lifestyle, post-diagnosis lifestyle changes

## Abstract

**Introduction:**

Colorectal cancer (CRC) is the third most common cancer worldwide and the second leading cause of cancer-related deaths. Nevertheless, it is estimated that more than 5.7 million people globally are currently living with it. CRC development is influenced by various environmental factors, and the benefits of a healthy lifestyle, particularly dietary changes, are receiving growing attention. While the mechanisms linking specific nutrients to CRC carcinogenesis are still under investigation, evidence suggests that prognosis and long-term outcomes may be influenced by post-diagnosis dietary habits.

**Methods:**

Here we report data from the ECHO 2.0 study (Eating habits CHanges in Oncologic patients), an observational investigation focused on post-diagnosis dietary changes, among Italian CRC patients. Data were collected from 98 subjects through a questionnaire designed to investigate changes in their food consumption, supplement use, and adoption of specific diets following surgery. Ninety-eight patients (45 females and 53 males) completed the survey, with most aged 65 years or older.

**Results:**

We observed that some patients reported positive dietary changes, including an increase in the consumption of vegetables, fresh fruit, and fish and shellfish, as well as a decrease in the intake of processed meat, desserts, red meat, animal fats, alcoholic beverages, refined bread and pasta, soft drinks, and baked goods. Despite CRC survivors made some positive changes to their nutritional habits, those modifications were mostly followed by a few of them, while over half of patients consumed nutritional supplements after diagnosis. Moreover, changes in nutritional habits were mainly adopted without consulting or informing the oncologist, and even if nearly half of patients believed that a moderate or strong link existed between cancer and nutrition, only one-third of them sought dietary information after being diagnosed.

**Discussion:**

Our findings suggest that, after diagnosis, CRC patients have made modest dietary changes, with only a few increasing their consumption of healthy foods. Furthermore, a significant proportion of survivors did not seek dietary information after their diagnosis, highlighting a gap in patient empowerment. These results emphasize the important role of healthcare providers, particularly oncologists, in identifying diet as a potential risk factor and directing patients toward appropriate nutritional support.

## 1 Introduction

An estimated 20 million new cancer cases and nearly 10 million cancer-related deaths occurred worldwide in 2022. These numbers are expected to rise by around 60% over the next two decades, with the global burden of cancer projected to surpass 35 million new cases and approximately 18.5 million cancer deaths by 2050 (1). Consequently, the continuous growth and aging of the world’s population, in conjunction with advances in early detection and treatment, have rendered cancer survivors a substantial component of the current population. In 2022, more than 53 million people worldwide were estimated to have survived five years after a cancer diagnosis (1).

Colorectal cancer (CRC) is the third most commonly diagnosed cancer, with more than 1.9 million cases, and the second leading cause of cancer-related deaths, with over 900,000 fatalities. However, improvements in treatment and the implementation of early diagnosis programs have steadily increased survival rates. It is currently estimated that there are more than 5.7 million people worldwide living with CRC (1).

Colorectal cancer has the second highest rate of incidence in the entire Italian population (12.6%), as well as in men (12.6%) and women (12.5%) (1). Nevertheless, Italy’s 5-year survival rate for CRC patients has increased from 52% in the 1990s to 65% for men and 66% for women today (2). Currently, 442,600 people in Italy have been diagnosed with colorectal cancer, including 227,600 men and 215,000 women (2).

Primary prevention has long been recognized as a critical strategy for reducing the global burden of cancer. Several studies have documented that dietary components and lifestyle behaviors are crucial modifiable factors in cancer risk. In fact, there is a growing body of evidence suggesting that 30-50% of cancers could be prevented by avoiding an unhealthy diet, physical inactivity, alcohol consumption, tobacco use, overweight, and obesity. Specifically, being overweight or obese is causally linked to the development of at least 13 different types of cancer, including CRC (3–10). Beyond the impact of body fatness, specific dietary components and physical activity are known to affect susceptibility to cancer development (3, 11). In agreement with that, many international organizations have established cancer prevention guidelines that highlight the importance of following a “healthy dietary pattern”, which is mainly based on the traditional Mediterranean nutritional model (3, 12–15). Although specific recommendations are still lacking, mounting evidence suggests that adhering to the same guidelines may be beneficial for cancer survivors, particularly in improving health-related quality of life (16–20).

Maintaining a healthy diet and lifestyle is especially crucial for preventing CRC. In fact, it appears that approximately 70% of new diagnoses are more influenced by environmental factors than genetic defects (6, 15, 21, 22). Key lifestyle factors associated with an increased risk of colorectal cancer include a diet high in red and processed meats, lack of physical activity, being overweight or obese, consuming alcohol, and smoking tobacco (6, 15, 21, 22). On the other hand, maintaining healthy lifestyle habits such as eating fiber-rich foods, consuming dairy products, keeping a healthy weight, and staying physically active can significantly reduce the risk of CRC, even for those with a higher genetic predisposition (6, 15, 21–23).

Despite the lack of definitive, large-scale epidemiological studies on CRC recurrence and survival outcomes, the World Cancer Research Fund (WCRF) recommends that colorectal cancer survivors follow general cancer prevention guidelines (3) to promote their overall health. Specifically, in 2024, the WCRF expert panel published a new report that summarizes the most recent research on diet, nutrition, physical activity and body weight, and which also provides advice for people living with and beyond colorectal cancer (17, 24). In summary, they suggest CRC survivors to consider: (i) following as many of WCRF/AICR’s cancer prevention recommendations (3) as they are able to; (ii) increasing their physical activity; (iii) increasing their whole grain intake; (iv) increasing their coffee intake; and (v) decreasing their sugary drink intake (17, 24). Accordingly, a very recent observational study showed that patients with stage III colon cancer who followed an anti-inflammatory diet (containing coffee, tea, dark yellow vegetables, and leafy greens) and exercised regularly had the best overall survival rates compared to those who exercised minimally and followed an inflammatory diet (comprising red meat, processed meats, refined grains, and sugary drinks) (25).

A cancer diagnosis creates a “teachable moment”, when patients and their families are highly motivated to adopt healthier lifestyles (26). A 2012 UK survey of 3,300 colorectal cancer patients found that over 20% were interested in receiving information about healthy eating and lifestyles following their diagnosis (27). Other publications have shown that these patients actively search the media for information on nutrition and health (28). In some cases, this resulted in an improved diet with increased consumption of fruits and vegetables (29, 30). Further studies show that 33-36% of patients change their eating habits after a colorectal cancer diagnosis, choosing healthier foods (whole grains, fruits, vegetables, and fish) while reducing alcohol, meat, and high-fat, sugar-sweetened meals. Additionally, the use of dietary supplements (mainly multivitamins and minerals) increased from 40% to over 50% following diagnosis (31–34). However, some recent research suggests that adherence to multiple lifestyle recommendations was low among colorectal cancer survivors, even though health behaviors have been reported to contribute to a higher quality of life among CRC patients (35, 36).

Although studies analyzing the effects of dietary habits on prognosis are increasing (37), little is still known about the current attitudes of colorectal cancer patients toward diet and nutrition. This lack of knowledge may hinder the development of personalized survivorship care approaches.

In 2021, we published the results of a prospective observational study examining changes in eating habits after a breast cancer (BC) diagnosis (38). The study was based on a sample of 684 patients who completed the ECHO (Eating Habits Changes in Oncologic Patients) Survey, a structured questionnaire designed specifically for the Italian population. Our findings revealed that most patients were interested in learning about the connections between lifestyle, diet, and cancer. However, few of them made dietary choices in line with national and international recommendations. Together with the fact that oncologists were rarely consulted for information or updates on dietary changes, this underscored the importance of fostering trust between patients and healthcare professionals and developing personalized counseling programs on proper nutrition and lifestyle (38).

Given our previous results and due to the limited number of studies on this topic, particularly those focusing on the Italian patient population with CRC, we propose an exploratory, prospective, observational study to investigate changes in dietary habits following a CRC diagnosis. To this end, the *ad hoc* ECHO (Eating habits CHanges in Oncologic patients) questionnaire 2.0 was used, which was minimally modified from the original version (38) to be adapted to the different survivors’ population.

Our primary aim was to explore and report on dietary changes and supplement use among patients with stage I-III colorectal cancer.

Then, we identified and discussed the sources of information most frequently accessed by CRC survivors that may have influenced the reported changes. Finally, we examined whether survivors had reported modifications in their dietary habits to their oncologist.

Treatment outcomes and quality of life for CRC patients may be improved by tertiary prevention strategies that focus on nutrition, ultimately aiming to prolong survival, support quality of life, and autonomy (39–41). We designed this study with the ultimate objective of generating hypotheses for future research and encouraging the development of personalized tertiary prevention strategies.

## 2 Materials and methods

### 2.1 Study design and patients

The ECHO (Eating habits CHanges in Oncologic patients) study 2.0 was conducted at the Medical Oncology Center of the IRCCS Sacro Cuore Don Calabria Hospital in Negrar di Valpolicella, Verona, Italy. The inclusion criteria were based on the following: (i) patients’ willingness to participate and general condition allowing understanding of the questions and purpose of the study; (ii) a histopathological diagnosis of stage I-III colorectal cancer, with surgery performed at least six months prior; (iii) a performance status of 0-1; (iv) normal organ and bone marrow function; and (v) no previous cancer diagnosis. The exclusion criteria included the following: (i) inability to answer questions; (ii) patients with permanent stomas or who, six months after surgery, have impaired digestive function; (iii) pregnancy or breastfeeding; (iv) clinically significant psychiatric, neurological, or medical disorders; and (v) a history of alcohol or drug abuse.

Patients were enrolled in the study at the moment of their first outpatient visits in the Department of Medical Oncology. A total of 100 patients were invited to participate in the study. Two patients declined the participation, resulting in a total number of 98 enrolled patients, all of whom were eligible. All enrolled patients who visited the medical center for treatment or follow-up were adults (over 18 years old) diagnosed with stage I-III colorectal cancer, and had undergone surgery at least six months prior to enrollment. All participants were required to sign an informed consent form. They were given all the necessary information in writing and were granted the unconditional and absolute right to withdraw at any time and for no specified reason.

Demographics, clinical characteristics, and histopathological features of patients are reported in **Tables 1** and **2**.

**Table 1:** Demographic and clinical characteristics of the overall patient sample (N = 98).

Characteristic	N = 98*^1^*
Time since diagnosis, months	
Median (Q1, Q3)	18.41 (12.62, 28.80)
Min, Max	0.79, 261.34
Missing	1
Age	
<65	39/97, 40.2%
>= 65	58/97, 59.8%
Missing	1
Sex	
Female	45/98, 45.9%
Male	53/98, 54.1%
Education	
Primary/Middle school	46/97, 47.4%
High school diploma	36/97, 37.1%
University degree	14/97, 14.4%
Master/PhD	1/97, 1.0%
Missing	1
Primary tumor site	
Colon	95/96, 99.0%
Rectum	1/96, 1.0%
Missing	2
Surgery	78/98, 79.6%
Antitumoral treatment received and/or ongoing:	
Chemotherapy	61/98, 62.2%
Experimental therapy	1/98, 1.0%
None	8/98, 8.2%

*^1^*n/N, %.

**Table 2:** Clinical and histopathological features of colon cancer patients (N = 95).

Colon cancer patients	N = 95*^1^*
Age	
18–34 years	1/95, 1.1%
35–49 years	3/95, 3.2%
50–64 years	34/95, 35.8%
65–79 years	46/95, 48.4%
> 80 years	11/95, 11.6%
Sex	
Female	44/95, 46.3%
Male	51/95, 53.7%
Weight (kg)	
Median (Q1, Q3)	71.00 (62.00, 82.00)
Min, Max	40.00, 143.00
Missing	1
Height (cm)	
Median (Q1, Q3)	168.00 (159.00, 175.00)
Min, Max	65.00, 196.00
Missing	1
BMI	
Underweight	3/94, 3.2%
Normal	44/94, 46.8%
Overweight	33/94, 35.1%
Obese	14/94, 14.9%
Missing	1
Performance Status (ECOG)	
0	76/94, 80.9%
1	18/94, 19.1%
Missing	1
**Surgery**	95/95, 100.0%
NO	0/95, 0,0%
YES	95/95, 100%
**Stoma**	6/94, 6.4%
Missing	1
Type of stoma	
Temporary	6/6, 100.0%
Permanent	0/6, 0.0%
Missing	89
Stoma site	
Ileostomy	6/6, 100.0%
Colostomy	0/6, 0.0%
Missing	89
Histology	
Adenocarcinoma	94/95, 98.9%
Other	1/95, 1.1%
Grading	
Unknown	6/95, 6.3%
G1	2/95, 2.1%
G2	64/95, 67.4%
G3	23/95, 24.2%
MSI/MMR	
Unknown	33/93, 35.5%
High	15/93, 16.1%
Low	45/93, 48.4%
Missing	2
KRAS	
Unknown	63/95, 66.3%
WT	21/95, 22.1%
Mutated	11/95, 11.6%
NRAS	
Unknown	68/95, 71.6%
WT	26/95, 27.4%
Mutated	1/95, 1.1%
BRAF	
Unknown	64/92, 69.6%
WT	17/92, 18.5%
Mutated	11/92, 12.0%
Missing	3
Tumor site:	
Right colon	50/95, 52.6%
Left colon	39/95, 41.1%
Transverse colon	6/95, 6.3%
Pathological stage	
I	8/94, 8.5%
IIA	35/94, 37.2%
IIB	4/94, 4.3%
IIC	4/94, 4.3%
IIIA	10/94, 10.6%
IIIB	22/94, 23.4%
IIIC	11/94, 11.7%
Missing	1
**Adjuvant therapy**	60/95, 63.2%
Type of adjuvant therapy	
Chemotherapy	60/60, 100.0%

*^1^*n/N, %.

### 2.2 The ECHO 2.0 questionnaire

The ECHO (Eating habits CHanges in Oncologic patients) 2.0 paper questionnaire was submitted to 98 patients with colorectal cancer from May 24, 2022 to November 4, 2025. The questionnaire was completed anonymously and administered by specialized medical oncologists who did not interfere with its completion. The oncologists were fully aware of the proper procedures and attitudes for approaching and treating patients with colorectal cancer, being able to address both the physical and emotional burdens of the disease.

Minor adjustments were made to the structure of the original questionnaire (38). Only a few section titles and response options regarding surgical and clinical treatments experienced by CRC survivors were modified. The questions patients answered remained unchanged from the original survey. The complete English translation is available as **Supplementary File 2** in the **Supplementary Material**. In brief, the survey consisted of 39 questions organized into four sections: (i) personal data and therapies, (ii) supplement use and specific diets followed after diagnosis, (iii) dietary changes after diagnosis, and (iv) beliefs about the relationship between diet and cancer. In the third section of the questionnaire, patients were asked to report whether their consumption of each of the 24 food items indicated (grouped into nine categories) had either remained the same, started, increased, decreased, stopped, or if a specific food had never been eaten. Moreover, the questionnaire investigated the sources of information used by the patients and whether any modifications in their dietary habits were reported to the oncologist, such as the use of supplements, dietary changes, or following a specific diet.

In order to perform a descriptive analysis of the hypothetical correlations existing among clinical and biopathological characteristics of the tumor, pharmacological treatments and dietary changes, the oncologist had to fill out a separate form for each assigned questionnaire (the full English translation is provided in **Supplementary File 1**). The form was used to record tumor biopathology and histology features, disease staging, and information about administered and/or current cancer treatments. The oncology form was standardized, and the data were extracted from medical records.

Once the ECHO 2.0 patient questionnaire, which was filled independently from the oncology form, was completed, it was anonymously submitted by the oncologist to the data manager. To ensure the confidentiality of their identities, each patient was assigned a unique identifier.

All the 98 collected questionnaires were provided with answers. Therefore, the final sample was represented by 98 colorectal cancer patients.

### 2.3 Statistical analysis

Categorical variables were summarized as absolute frequencies and percentages, while continuous variables were described using medians and interquartile ranges (Q1–Q3). Changes in food consumption after CRC diagnosis were analyzed by categorizing responses as “no change” (including same or never eaten) versus “change” (including started, increased, decreased, or stopped consumption). Comparisons between age groups (<65 years vs. ≥65 years), educational level, and nutritional supplement used were performed using Pearson’s chi-squared test or Fisher’s exact test, as appropriate based on expected cell counts. Differences in the number of supplements used were assessed using the Wilcoxon rank-sum test. Statistical significance was set at a two-sided p-value <0.05. All analyses were conducted using standard statistical software.

## 3 Results

### 3.1 Changes in food consumption after CRC diagnosis

**Figure 1:**
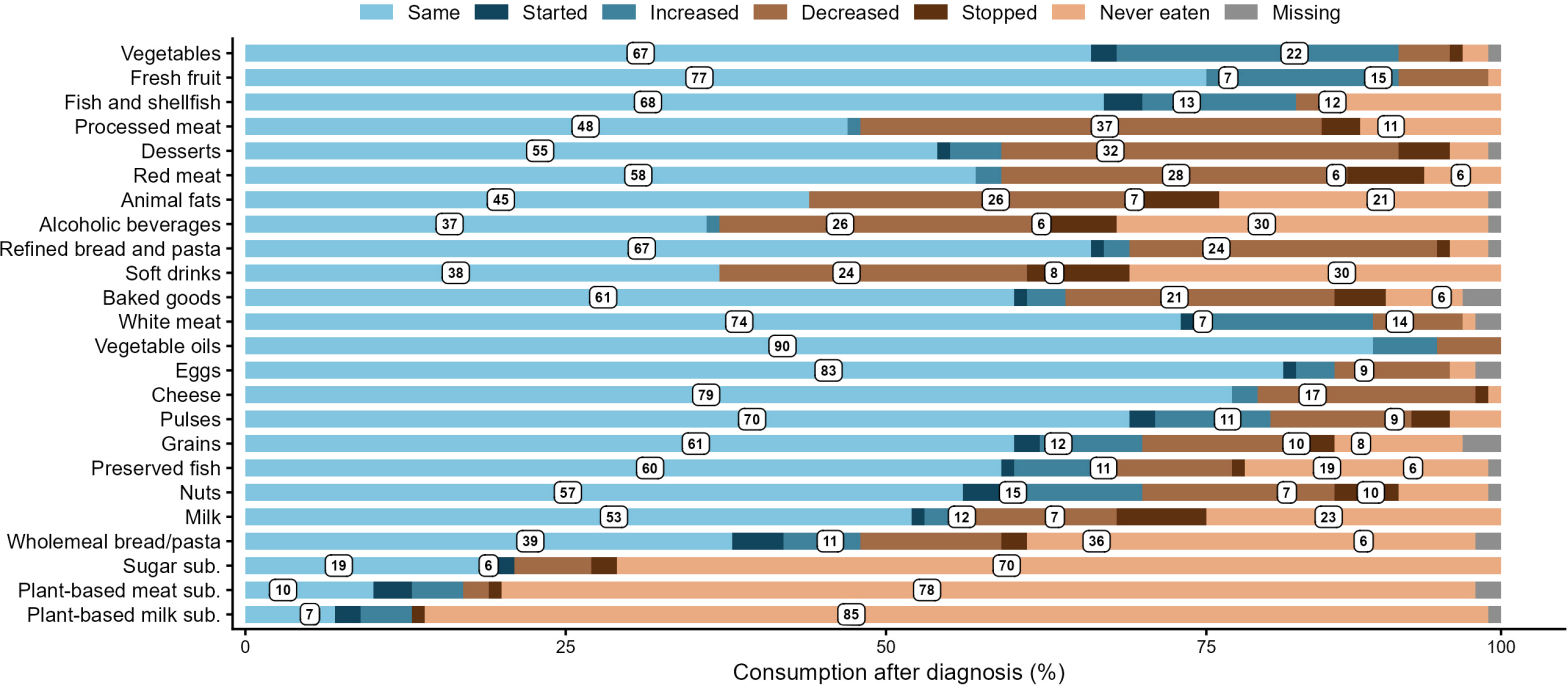
Changes in food consumption after colorectal cancer diagnosis. Respondents’ reported food consumption patterns are expressed as percentages. “Missing” indicates that no responses were provided.

In order to investigate whether any changes in dietary habits were reported by patients, they were asked to state if and how their consumption of 24 specific food items varied or not after CRC diagnosis. As illustrated in **Figure 1**, in line with WCRF recommendations, 22%, 15%, and 12% of survivors increased their intake of vegetables, fresh fruit, and fish and shellfish, respectively (**Figure 1**). Consistent with cancer prevention guidelines, patients decreased their consumption of processed meat (37%), desserts (32%), red meat (26%), animal fats (26%), alcoholic beverages (26%), refined bread and pasta (24%), soft drinks (24%), and baked goods (21%) (**Figure 1**). Additionally, 14% of patients increased their consumption of white meat (**Figure 1**). There were no significant variations in the intake of vegetable oils, eggs, cheese or pulses (**Figure 1**).

Individuals under 65 years of age were more likely to experience a change in their consumption of vegetables, and whole wheat bread and grains (**Table 3**). In particular, the change in food consumption among those under 65 was characterized by a significant increase in vegetable and whole wheat bread and grain consumption (**Table 3**).

**Table 3:** Changes in consumption of vegetables, whole wheat bread, and grains according to age group (<65 vs ≥65 years).

Characteristic	Age		p-value*^2^*
	<65 N = 39*^1^*	>= 65 N = 58*^1^*	
Whole-grain bread and pasta			**0.019**
No change	24/38, 63.2%	48/57, 84.2%	
I changed the way I consume	14/38, 36.8%	9/57, 15.8%	
Missing	1	1	
Whole-grain bread and pasta			**0.017**
Consumption unchanged	15/38, 39.5%	23/57, 40.4%	
Started consuming	2/38, 5.3%	2/57, 3.5%	
Increased consumption	6/38, 15.8%	0/57, 0.0%	
Reduced consumption	5/38, 13.2%	6/57, 10.5%	
Stopped consuming	1/38, 2.6%	1/57, 1.8%	
Never consumed	9/38, 23.7%	25/57, 43.9%	
Missing	1	1	
Vegetables			0.10
No change	24/39, 61.5%	44/57, 77.2%	
I changed the way I consume	15/39, 38,5%	13/57, 22.8%	
Missing	0	1	
Vegetables			**0.021**
Consumption unchanged	23/39, 59.0%	43/57, 75.4%	
Started consuming	1/39, 2.6%	1/57, 1.8%	
Increased consumption	14/39, 35.9%	7/57, 12.3%	
Reduced consumption	0/39, 0.0%	4/57, 7.0%	
Stopped consuming	0/39, 0.0%	1/57, 1.8%	
Never consumed	1/39, 2.6%	1/57, 1.8%	
Missing	0	1	

*^1^*n/N, %.

*^2^*Fisher’s exact test, Pearson’s Chi-squared test.

“I changed the way I consume” corresponds to: “Started consuming” + “Increased consumption” + “Reduced consumption” + “Stopped consumption”; “Missing” indicates that no responses were provided. Statistically significant results are reported in bold.

### 3.2 Nutritional supplements intake and diets followed, after CRC diagnosis

**Figure 2:**
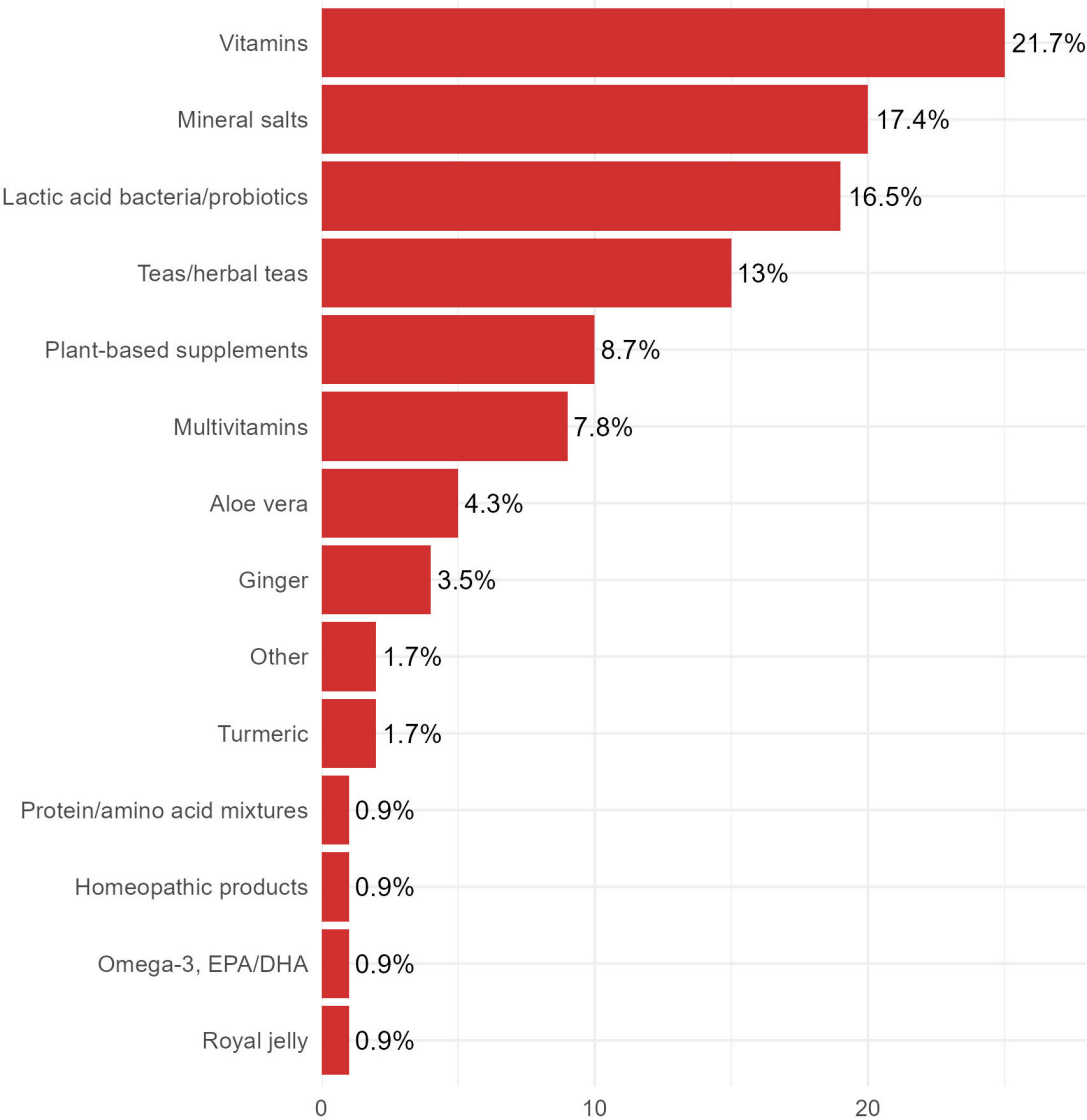
Use of supplements after colorectal cancer diagnosis. Respondents could select more than one answer.

We also investigated whether our sample population of CRC survivors started using supplements or following specific diets after cancer diagnosis, as the literature frequently details (32, 42–44). Notably, more than 50% of patients reported having consumed a nutritional supplement at least once (**Table 4**). These survivors mainly mentioned consuming vitamins (almost the 22%), followed by mineral salts (17.4%), lactic acid bacteria/probiotics (16.5%), teas/herbal teas or infusions (13%), plant-based supplements (8.7%), and multivitamins (7.8%). Aloe vera and ginger were used by the 4.3% and 3.5% of this sample, respectively (**Figure 2**). The remaining products, including turmeric, protein/amino acid mixtures, homeopathic products, and omega-3, EPA/DHA, were chosen by 1.7%, or less, of the survivors (**Figure 2**).

**Table 4:** Attitudes toward using nutritional supplements, after colorectal cancer diagnosis.

Characteristic	N = 98*^1^*
Age	
18–34 years	1/98, 1.0%
35–49 years	3/98, 3.1%
50–64 years	36/98, 36.7%
65–79 years	47/98, 48.0%
> 80 years	11/98, 11.2%
Sex	
Female	45/98, 45.9%
Male	53/98, 54.1%
I started using AT LEAST one product after diagnosis	
No	45/98, 45.9%
Yes	53/98, 54.1%
**Reason for using these products: nutritional deficiencies**	12/98, 12.2%
**Reason for using these products: support for weight loss**	4/98, 4.1%
**Reason for using these products: support during chemotherapy/radiotherapy**	17/98, 17.3%
**Reason for using these products: to fight cancer**	0/98, 0.0%
**Reason for using these products: other**	18/98, 18.4%
**Who suggested these products or provided information?: self-directed (books/internet/seminars)**	14/98, 14.3%
**Who suggested these products or provided information?: general practitioner**	21/98, 21.4%
**Who suggested these products or provided information?: oncologist**	3/98, 3.1%
**Who suggested these products or provided information?: dietitian/nutritionist**	6/98, 6.1%
**Who suggested these products or provided information?: pharmacist**	9/98, 9.2%
**Who suggested these products or provided information?: naturopath/homeopath/other non-conventional practitioners**	1/98, 1.0%
**Who suggested these products or provided information?: family members, friends, relatives**	7/98, 7.1%
**Who suggested these products or provided information?: patients with the same condition**	0/98, 0.0%
**Did you inform your oncologist about the use of these products?**	15/51, 29.4%
Missing	47

*^1^*n/N, %. Statistically significant results are reported in bold.

As shown in **Table 4**, the primary reason for CRC survivors to use food supplements was to mitigate the adverse effects of therapies (17.3%), followed by addressing nutritional deficiencies (12.2%), and assisting with weight loss (4.1%).

The 21.4% of the patients who started to consuming nutritional supplements stated that they followed the general practitioner’s (GP) medical recommendation, while the 14.3% of them made those decisions relying on their own research from the Internet, books, or seminars (**Table 4**). Only 52% of patients answered the question about whether they had informed their oncologist about their supplement use. Of those who responded, only 29.4% answered “yes” (**Table 4**).

As shown in **Table 5**, we found that people under 65 were significantly more likely to take a food supplement after their diagnosis. The main reasons for CRC survivors in this age group to do so were to counteract therapy side effects (33.3%, p-value <0.001) and to lose weight (10.3%, p-value 0.024) (**Table 5**). The multivariate logistic regression analysis also revealed that 20% (p-value 0.021) of people with a higher education took food supplements to counteract nutritional deficiencies (**Table 5**). Finally, 15.4% (p-value 0.003) of survivors under 65 years of age stated that they started taking supplements following dietitians’ and nutritionists’ recommendations (**Table 5**).

**Table 5:** Use of nutritional supplements according to age group (<65 vs ≥65 years) and education (High school diploma/University degree vs Primary/Middle school).

Characteristic	Age		p-value*^2^*
	<65 N = 39*^1^*	>= 65 N = 58*^1^*	
How many products did you start using after diagnosis?			0.013
Median (Q1, Q3)	2.00 (0.00, 2.00)	0.00 (0.00, 1.00)	
Min, Max	0.00, 5.00	0.00, 8.00	
I started using AT LEAST one product after diagnosis			0.018
No	12/39, 30.8%	32/58, 55.2%	
Yes	27/39, 69.2%	26/58, 44.8%	
**Reason for using these products: support for weight loss**	4/39, 10.3%	0/58, 0.0%	**0.024**
**Reason for using these products: support during chemotherapy/radiotherapy**	13/39, 33.3%	4/58, 6.9%	**<0.001**
**Who suggested these products or provided information?: dietitian/nutritionist**	6/39, 15.4%	0/58, 0.0%	**0.003**
	Education		
	High school diploma/University degree N = 50*^1^*	Primary/Middle school N = 46*^1^*	p-value*^2^*
**Reason for using these products: nutritional deficiencies**	10/50, 20.0%	2/46, 4.3%	**0.021**

*^1^*n/N, %.

*^2^*Wilcoxon rank sum test; Pearson’s Chi-squared test; Fisher’s exact test. Statistically significant results are reported in bold.

Of the 98 patients diagnosed with CRC, only 15 followed a specific diet. Among them, 4.1% wanted to lose weight, and 3.1% sought to mitigate the adverse effects of therapy (**Supplementary Table 1**). Even if the survivors started new diets based on GP recommendations or the advice of dietitians and nutritionists, the vast majority of them (70%) did not inform their oncologists (**Supplementary Table 1**).

### 3.3 Patients’ beliefs and information needs regarding nutrition and cancer

The final three questions of the survey aimed to determine CRC survivors’ beliefs about the relationship between nutrition and cancer and their informational needs on the topic. Interestingly, we found that nearly half of patients believes that a moderate or strong link between cancer and nutrition does exist. Specifically, this feeling was reported by the 81.3% of respondents aged below 65 years and the 55.3% of patients over 65 years. In contrast, only the 18.8% of the youngest believed that there is little or no connection between cancer and nutrition, compared to the 44.7% of the respondents aged above 65 years (**Table 6**).

**Table 6:** Beliefs about cancer and nutrition.

Characteristic	Age		p-value*^2^*
	<65 N = 39*^1^*	>= 65 N = 58*^1^*	
**Do you believe that diet can be linked to oncological diseases?**			**0.021**
No/A little	6/32, 18.8%	17/38, 44.7%	
Quite a bit/A lot	26/32, 81.3%	21/38, 55.3%	
Missing	7	20	
**After diagnosis, did you ask anyone for information about diet?**	19/39, 48.7%	14/58, 24.1%	**0.012**
**Who provided information?: self-directed (books/internet/seminars)**	7/39, 17.9%	0/58, 0.0%	**0.001**
**Who provided information?: general practitioner**	6/39, 15.4%	9/58, 15.5%	>0.9
**Who provided information?: oncologist**	4/39, 10.3%	6/58, 10.3%	>0.9
**Who provided information?: dietitian/nutritionist**	10/39, 25.6%	2/58, 3.4%	**0.003**
**Who provided information?: pharmacist**	0/39, 0.0%	0/58, 0.0%	>0.9
**Who provided information?: naturopath/homeopath/other non-conventional practitioners**	0/39, 0.0%	0/58, 0.0%	>0.9
**Who provided information?: family members, friends, relatives**	1/39, 2.6%	1/58, 1.7%	>0.9
**Who provided information?: patients with the same condition**	0/39, 0.0%	1/58, 1.7%	>0.9

*^1^*n/N, %.

*^2^*Fisher’s exact test; Pearson’s Chi-squared test. Statistically significant results are reported in bold.

After receiving a CRC diagnosis, nearly 34% of respondents asked someone for information about nutrition. Of those, 48.7% were younger than 65 years old, while 24.1% were over 65 years old (**Table 6**). The main sources of information for the youngest survivors were dietitian/nutritionist (25.6%), followed by their own research (the Internet, books, or seminars) (17.9%), the GP (15.4%), and the oncologist (10.3%); while patients over 65 years of age mainly relied on the general practitioner (15.5%) and the oncologist’s (10.3%) opinion. None of them would use the Internet, books, or seminars as sources of information (**Table 6**).

## 4 Discussion

Interest in nutrition and dietary changes often accompanies a cancer diagnosis, particularly in the case of breast cancer (38, 45). This is probably due to a number of reasons, including the fact that BC survivors (mostly women) make up the largest group within the survivor community (1). Additionally, compared to men, women have stronger beliefs in healthy eating and actively seek out nutrition information, which suggests that they may be more likely to make dietary changes when facing a health condition (38, 45, 46). Finally, many of those patients are very active in the online support community, where they share their experiences and offer support to others, even regarding dietary choices (47).

Compared to BC survivors, fewer studies have been conducted on how and why patients with colorectal cancer make dietary decisions after diagnosis. However, since a healthy diet and lifestyle are essential for preventing and treating colorectal cancer (6, 15, 17, 21–25), it is crucial to understand the nutritional choices of these patients after they are diagnosed. This knowledge can help guide them toward making informed, healthy decisions.

In this study, we aimed to explore changes in eating habits and supplement use among a group of Italian colorectal cancer survivors. We also wanted to identify the main sources of information that may have affected those changes and determine whether the physician was informed of the modifications.

Previous publications showed that CRC patients made some dietary changes since the time of diagnosis; however, the results were mixed. A longitudinal study of patients in the Netherlands with stage I to III colorectal cancer, by Van Zutphen M et al. (48), demonstrated that only minor changes were made. Specifically, survivors decreased their intake of sugary drinks and red and processed meat but did not change their consumption of fruits, vegetables, alcohol, or ultra-processed foods (48). Another investigation analyzing changes in the dietary habits of an Italian group of patients with different types of tumors found that 61.1% of respondents who reported a nutritional change were CRC survivors (49). A study of various cancer types in the United States found that 15.5% of patients diagnosed with colorectal cancer reported at least one dietary change within the previous year (31). Other research has shown that between 33% and 36% of patients have reported improvements in their diet, including increased consumption of fruits and vegetables, whole grains, and fish, as well as reduced intake of alcohol, meat, and foods high in fat, sugar, and salt (29, 30). However, recent studies indicate that many colorectal cancer survivors are not following multiple lifestyle recommendations, even though behaviors like smoking cessation, exercise, and consuming healthy foods have been shown to improve quality of life (35, 36). Consistent with these data, we observed that our group of survivors made some changes that partially aligned with the WCRF recommendations (3), reporting increased consumption of vegetables, fresh fruit, fish, and shellfish, as well as decreased intake of processed meat, desserts, red meat, animal fats, alcohol, refined bread and pasta, soft drinks, and baked goods. However, fewer than half of the overall sample made the correct dietary modifications. Nevertheless, we mainly observed a trend toward reducing consumption of foods and beverages associated with an increased risk of CRC, such as processed and red meat and alcohol. Also, the intake of desserts, animal fats, soft drinks, and baked goods, whose excessive consumption increases the risk of overweight and obesity and, thus, indirectly, of colorectal cancer, was diminished. Notably, unlike the results reported by Van Zutphen M et al. (48), the intake of vegetables and fresh fruit was increased, augmenting the ingestion of fiber, which is another habit reported to be beneficial for gut health and CRC prevention. Although we did not observe any significant variations in pulse or dairy product consumption, we can hopefully hypothesize that the previously described choices were made intentionally to reduce the risk of CRC recurrence and improve survival outcomes.

Importantly, we found that people under 65 were significantly more likely to increase their consumption of vegetables, whole wheat bread, and grains compared to older patients. On the contrary, not only survivors over 65 years of age did not increase their consumption of these foods, but nearly 44% of them declared that they had never eaten whole wheat bread or grains. These results may suggest that younger patients have a higher level of health literacy, possibly due to better education and greater Internet and social network use. The latter are often sources of health-related information and are more familiar to younger respondents (45, 50, 51). This result aligns with some of the findings from our previous publication, in which we observed that older survivors were more likely to maintain their dietary habits (38). As outlined previously (38), our latest findings reinforce the suggestion to primarily identify precise nutritional interventions for specific patient categories, such as older survivors, as they may experience more difficulty initiating and/or maintaining nutritional changes on their own.

Next, we analyzed whether this population of CRC survivors began to use supplements or follow particular diets after their cancer diagnosis. Previous studies have reported increased use of dietary supplements among many survivor populations (43, 52–54). Regarding colorectal cancer patients, the use of dietary supplements (mainly multivitamins and minerals) has been reported to increase from 40% to over 50% following diagnosis (31–34). Consistent with this, more than 50% of our respondents reported having consumed a nutritional supplement at least once, with the most common options being vitamins, mineral salts, lactic acid bacteria/probiotics, and tea, herbal tea or infusions. As previously reported (32, 55), they stated that the main reasons for taking supplements were to counteract the side effects of therapies, followed by addressing nutritional deficiencies.

Interestingly, people under the age of 65 were significantly more likely than older patients to begin taking a dietary supplement after receiving a diagnosis. Consistent with previous studies (32, 55), the primary reasons for CRC survivors in this age group to take supplements were to counteract therapy side effects and, to a lesser extent, to lose weight. After taking the level of education into account, we found that 20% of the most highly educated respondents took food supplements to counteract nutritional deficiencies, compared to 4% of the less educated survivors. This could possibly indicate a major interest in health among better-educated people compared to individuals who had less formal schooling (56).

In contrast to the consumption of supplements, the adherence to a specific diet after diagnosis was extremely low, with only 15.3% of the survivors in our sample declaring that they had followed one. The two main reasons driving patients to adopt a new diet were weight loss and mitigation of the adverse effects of therapy, which are the same motivations given for dietary supplement use. Notably, nearly 50% of our respondents reported being overweight or obese, conditions associated with an increased risk of CRC (3–10) and possibly its recurrence (17, 24). Moreover, some studies have shown that patients undergoing colorectal cancer surgery tend to gain weight (57–59). Although the evidence regarding the association between weight change and survival after a colorectal cancer diagnosis (particularly a metastatic one) is controversial (60), we can hypothesize that some of our survivors may have been interested in achieving a healthy body weight to improve their prognosis.

As previously reported (28, 38), most patients who began taking nutritional supplements or following a diet had not received professional advice about it. Fewer than 22% of our survivors followed their general practitioner’s medical recommendation, while 14% made these decisions based on their own research from the Internet, books, or seminars. Furthermore, only 15% and 7% of patients, respectively, informed their oncologist about taking supplements or beginning new diets. Interestingly, the same percentage of respondents who told the oncologist about starting a specific diet received information on the subject from a healthcare professional, such as a GP or a dietitian/nutritionist.

When all this is taken into account, it might be advisable for clinicians to regularly ask patients about their supplement use and to suggest caution due to a lack of evidence for any beneficial effects and, instead, of potential increased risks, in terms of cancer recurrence and harmful interaction with therapies (53, 61). Furthermore, since nutritional information from health professionals has been shown to influence survivors’ beliefs about the impact of nutrition on cancer outcomes and help CRC patients maintain good health and quality of life over time (20, 55), oncologists should be open to questions on this topic and ready to counsel patients on the appropriate use of dietary supplements.

Earlier studies have shown that some CRC patients have an interest in receiving information about healthy eating and lifestyles, and sometimes they actively search the media for nutrition and health advice (27, 28, 49). The final three questions of the survey were designed to determine how CRC survivors believe nutrition relates to cancer and what information they need on the topic. Interestingly, nearly half of the surveyed patients believe that there is a moderate or strong link between cancer and nutrition. Specifically, over 80% of respondents under 65 years old reported this, as did nearly 55% of those over 65. In contrast, fewer than 20% of the youngest survivors felt there was little or no link, compared to nearly 45% of those over 65. Similarly to our previous publication on breast cancer patients (38), the sources of information used most frequently by the youngest survivors to gain insight on tumor risk and nutrition were dietitians/nutritionists, followed by their own research. In contrast, patients over 65 years of age mainly relied on general practitioners and oncologists, and did not consider using the Internet, books, or seminars as sources of information. These results support the above-mentioned possibility that our youngest respondents have higher health literacy and greater confidence in using the Internet and social media. However, the fact that patients over 65 rely on health professionals could be valuable, as it opens up the opportunity for them to be more willing to follow clinicians’ dietary and lifestyle advice.

Overall, our survey data indicate that only a minority of CRC survivors were aware of a few dietary messages and made positive changes to their nutritional habits. Even so, they did not fully comply with the cancer prevention nutritional guidelines (3).

As was previously observed with BC patients (38), CRC survivors over the age of 65 were considerably less interested in changing their eating habits than younger patients. They also seemed less aware of the existing link between nutrition and cancer, even if they could be more open to the advice of clinicians. Furthermore, we found that most patients took nutritional supplements after being diagnosed without first consulting or informing their oncologist.

Since some CRC patients, especially those under 65 years of age, searched for information in the general media or online, it is possible that they also encountered incorrect or misleading nutritional messages. Considering that many survivors search the Internet for information on cancer and nutrition because they find it difficult to get dietary advice from their oncologists (47, 62–64), improving the physician-patient relationship is a crucial point. This will enable health professionals to better address survivors’ needs and ultimately provide customized nutrition counseling and lifestyle intervention programs throughout the cancer journey.

Two main strengths of this study include the use of a validated and tested questionnaire (38) and the survey administration method. The use of a tested survey results in a decrease in sampling error and an increase in final response rates, leading to more accurate data and better representation of the target population. Additionally, self-reported data have been shown to reduce interviewer bias and encourage truthful responses. Finally, our questionnaire might provide the opportunity to perform a descriptive analysis of the hypothetical correlations existing among clinical histopathological, and biopathological characteristics of the tumor, pharmacological treatments and dietary changes, as described in the “Patients and Methods” section. However, some limitations should be kept in mind. Even if the questionnaire has been pre-tested and successfully used in a previous publication (38), neither the current study nor the previous ones involved psychometric validation. Additionally, our sample size was quite small, which limited the generalizability and the statistical power of certain results. Moreover, since the survey was self-reported, patients may have inaccurately recalled their nutritional habits and/or overemphasized healthier food choices, as this is more socially desirable. Also, since we used a qualitative approach to explore eating behaviors, we lack quantitative measurements of food consumption, thus limiting the report of a detailed dietary analysis. Furthermore, we did not analyze or compare dietary habits before and after diagnosis. Finally, although we identified a group of patients over the age of 65 who could be primary candidates for personalized lifestyle interventions, our questionnaire did not explore factors besides age and basic education that might influence dietary behaviors, such as systemic barriers, psychological factors, cultural factors, and socioeconomic status.

## 5 Conclusion

Importantly, our findings advance the understanding of dietary needs and behaviors of colorectal cancer survivors, and also help identify patient categories, such as those over 65 years old, who could be primary candidates for personalized lifestyle interventions. Moreover, this study reinforced the crucial role of healthcare providers, particularly oncologists, in addressing diet as a potential risk factor and providing patients with appropriate nutritional support.

Although preliminary, these findings could help to structure future tertiary nutritional interventions for colorectal cancer patients. In fact, the ECHO Survey 2.0 offers the chance to apply to a broader group of CRC survivors, even in countries beyond Italy, with the aim of gaining deeper understanding about their dietary habits, information needs, and beliefs about the link between diet and cancer. Notably, the survey proved capable of identifying specific categories of survivors with unique habits, knowledge, and needs. These kinds of insights might be crucial to assist in the development of informed, tailored nutritional interventions that could encourage patients to adhere to specific recommendations. As earlier research has suggested (65), in fact, these initiatives have the capacity to encourage cancer survivors to comply more closely to nutritional and lifestyle recommendations, which can ultimately prevent recurrence and improve life expectancy.

## Data Availability

The original contributions presented in the study are included in the article/**Supplementary Material**. Further inquiries can be directed to the corresponding author.
