# Supplementary material for: Changes in dietary habits following a colon cancer diagnosis: insights from the prospective ECHO (Eating habits CHanges in Oncologic patients) STUDY 2.0
Source: Front Oncol. 2026 May 21;16:1843736. doi: 10.3389/fonc.2026.1843736 (PMC13233392; doi:10.3389/fonc.2026.1843736)
Supplement: Supplementary file 1 [file DataSheet1.docx]

Supplementary Material

# Supplementary Data

**SUPPLEMENTARY FILE 1**. Oncologist form.

**PERSPECTIVE OBSERVATIONAL STUDY ON CHANGES IN DIETARY HABITS AFTER COLORECTAL CANCER DIAGNOSIS (ECHO STUDY 2.0)**

CENTER CODE #______

PATIENT CODE #______

MONTHS SINCE SURGERY:

#______

Date of form completion___/___/_____

**Date of birth**___/___/_____

**Gender**: ☐ F ☐ M

**Weight**: (kg)____**Height**: (cm)____**PS** (ECOG)____

**Date of first diagnosis** (cytology/histology) ___/___/_____

**Histotype**: ☐ (Specify)_________________________

**Grading**: ☐ G1 ☐ G2 ☐ G3 ☐ Unknown

**MSI/MMR**: ☐ High ☐ Low ☐ Unknown

**KRAS**: ☐ WT ☐ Mutated ☐ Unknown

**NRAS**: ☐ WT ☐ Mutated ☐ Unknown

**BRAF**: ☐ WT ☐ Mutated ☐ Unknown

**Location**: ☐ Rectum ☐ Right colon ☐ Left colon ☐ Transverse colon

**Neoadjuvant treatment:**

☐ **NO** ☐ **YES**

If **YES**: **Start Date** ___/___/_____

**Type**: ☐ Radiation therapy ☐ Chemotherapy ☐ Chemotherapy + radiation therapy ☐ Clinical study ☐ Other (Specify)______________________

**Chemotherapy**: ☐ Capecitabine ☐ Xelox ☐ Folfox

**Clinical Status: cT___________ cN___________ M0___________**

**Surgery**

**Weight**: (kg)____ before surgery

Surgery: ☐ **NO** ☐**YES**

If **YES**: **Date**__/___/____; **Type:** ________________

**Stoma**: ☐ **NO** ☐ **YES**

If **YES**: **Date**__/___/____; **Type**: ☐ Temporary ☐ Permanent;

**Location**: ☐ Ileostomy ☐ Colonostomy

**Post-operative biological and molecular parameter status:**

**Histotype:** ☐ (Specify)_________________________

**Grading:** ☐ G1 ☐ G2 ☐ G3 ☐ Unknown

**MSI/MMR**: ☐ High ☐ Low ☐ Unknown

**KRAS**: ☐ WT ☐ Mutated ☐ Unknown

**NRAS**: ☐ WT ☐ Mutated ☐ Unknown

**BRAF**: ☐ WT ☐ Mutated ☐ Unknown

**Location**: ☐ Rectum ☐ Right colon ☐ Left colon ☐ Transverse colon

**Pathological status:** ☐ **IA** ☐ **IIA** ☐ **IIB** ☐ **IIC** ☐ **IIIA** ☐ **IIIB** ☐ **IIIC**

**Adjuvant therapy:**

☐ **NO** ☐ **YES**

If **YES**: **Start Date** ___/___/_____

**Type**: ☐ Radiation therapy ☐ Chemotherapy ☐ Chemotherapy + radiation therapy ☐ Clinical study ☐ Other (Specify)______________________

**Chemotherapy**: ☐ Capecitabine ☐ Xelox ☐ Folfox

**Clinical Status: cT___________ cN___________ M0___________**

**Outcome:**

**Recurrence**: ☐ **NO** ☐ **YES**

If **YES**: **Date**__/___/____

**Location**: ☐ Locoregional ☐ Distant metastasis

**SUPPLEMENTARY FILE 2**. The “ECHO” (Eating habits CHanges in Oncologic patients) 2.0 questionnaire.

CENTER CODE #______

PATIENT CODE #______

Date_____________

# ‘ECHO’ QUESTIONNAIRE 2.0

Dear patient,

With the following questionnaire, we aim to add to our current data so as to more accurately identify changes in patients’ dietary habits, after cancer treatment, meaning at least 6 months after surgery. Our intention is to evaluate how frequently, and to what degree, changes in diet occur and to identify the information sources most often used.

The questionnaire is simple to complete and will only take a few minutes of your time. It is completely anonymous.

How to fill in the form:

Answer each question in the questionnaire by ticking the relevant answer. If you are uncertain, choose the answer that fits best.

Please tick one answer only out of those listed, unless the question itself allows for more than one option (*“more than one answer possible”*).

Bear in mind that this questionnaire is not an evaluation and there are no right or wrong answers.

We ask you to answer the questions personally. Please do not get your partner, relatives, friends or visitors to help you in the completion of this questionnaire.

Thank you for taking the time to complete this questionnaire for us.

**ECHO QUESTIONNAIRE 2.0**

| **PERSONAL DETAILS AND CANCER THERAPIES** |
| --- |

| 1. Age |  | 〇18-34 years  〇35-49 years  〇50-64 years  〇65-79 years  〇 > 80 years |
| --- | --- | --- |
| 2. Gender |  | 〇 F  〇 M |
| 3. Level of Education |  | 〇 Primary/Middle School  〇 High School  〇 University Degree  〇 Master’s/PhD |
| 4. Cancer treatments – finished and/or ongoing  *(****more than one answer possible****)* |  | ☐ None  ☐ Surgery  ☐ Radiotherapy  ☐ Chemotherapy  ☐ Experimental therapy |

| **VITAMINS, OTHER SUPPLEMENTS AND SPECIFIC DIETS AFTER ONCOLOGICAL THERAPIES**  **(At least six months after surgery)** |
| --- |

| 5. Did you begin to use one or more of these products following your diagnosis?  *(****more than one answer possible****)* | ☐ vitamins (eg vit. C, vit. D)  ☐ mineral salts (eg calcium, potassium, magnesium)  ☐ multivitamins  ☐ omega-3, EPA/DHA  ☐ plant-derived supplements (tablets/capsules/drops)  ☐ lactic cultures/probiotics  ☐ homeopathic products  ☐ soy phytoestrogens  ☐ protein powder/amino acids  ☐ tea/herbal tea/infusions  ☐ royal jelly  ☐ aloe vera  ☐ reishi mushroom  ☐ açai fruit  ☐ goji berries  ☐ turmeric  ☐ ginger  ☐ other *(please specify)*_________________ |
| --- | --- |
| 5.1. If your answer was YES, why have you used these products?  (***more than one answer possible)*** | ☐ a nutritional deficiency  ☐ help to lose weight  ☐ help during chemo/radiotherapy  ☐ to fight cancer  ☐ other *(please specify)*_________________ |
| 5.2. If your answer was YES, who suggested the use of these products or gave you information about them?  *(****more than one answer possible****)* | ☐ myself using literature/Internet/ seminars  ☐ general practitioner (GP)  ☐ oncologist  ☐ dietician/nutritionist  ☐ pharmacist  ☐ naturopathic doctor/homeopathic doctor/other non-conventional medical practitioner  ☐ family/friend  ☐ patients with the same medical condition |
| 5.3. If your answer was YES, have you spoken with your oncologist about the products you take? | 〇 Yes  〇 No |

| 6. If you started a specific diet after your diagnosis, which type of diet was it?  *(****more than one answer is possible****)* | ☐ vegetarian diet  ☐ vegan diet  ☐ macrobiotic diet  ☐ high-protein diet  ☐ blood type diet  ☐ alkaline diet  ☐ glycemic index diet  ☐ paleo diet  ☐ raw food diet  ☐ detox diet/based on fruit and/or vegetable juices  ☐ Kousmine method diet  ☐ fasting mimicking diet  ☐ other *(please specify)*_________________ |
| --- | --- |
| 6.1. If you started a specific diet, what were your reasons for doing so?  *(****more than one answer is possible****)* | ☐ a nutritional deficiency  ☐ help to lose weight  ☐ help during chemo/radiotherapy  ☐ to fight cancer  ☐ other *(please specify)*_________________ |
| 6.2. If you started a specific diet, who suggested or gave you information about the diet?  *(****more than one answer possible****)* | ☐ myself using literature/Internet/ seminars  ☐ general practitioner (GP)  ☐ oncologist  ☐ dietician/ nutritionist  ☐ pharmacist  ☐ naturopathic doctor/homeopathic doctor/other non-conventional medical practitioner  ☐ family or friends  ☐ patients with same medical condition |
| 6.3. If you started a specific diet, have you spoken with your oncologist about the diet you started? | 〇 Yes  〇 No |

| **EATING HABITS AFTER ONCOLOGICAL THERAPIES**  **(At least six months after surgery)** |
| --- |

| ***CERALS AND CERALS PRODUCTS***  *How have your eating habits changed* ***since your diagnosis****?*  *(Only one answer is possible for each question)*  **7. WHOLEMEAL BREADAND PASTA 8. REFINED BREAD AND PASTA**  〇 eat the same 〇 eat the same  〇 have started eating 〇 have started eating  〇 eat more 〇 eat more  〇 eat less 〇 eat less  〇 no longer eat 〇 no longer eat  〇 have never eaten 〇 have never eaten  **9. BAKED GOODS 10. GRAINS**  **(crackers, breadsticks, biscuits, cakes, etc.) (rice, spelt, barley, quinoa, buckwheat, amaranth, etc.)**  〇 eat the same 〇 eat the same  〇 have started eating 〇 have started eating  〇 eat more 〇 eat more  〇 eat less 〇 eat less  〇 no longer eat 〇 no longer eat  〇 have never eaten 〇 have never eaten |
| --- |

| **EATING HABITS AFTER ONCOLOGICAL THERAPIES**  **(At least six months after surgery)** |
| --- |

| ***FRUIT, VEGETABLES, PULSES***  *How have your eating habits changed* ***since your diagnosis****?*  *(Only one answer is possible for each question)*  **11. FRESH FRUIT 12. NUTS**  **(walnuts, hazelnuts, almonds, pistachios, etc.)**  〇 eat the same 〇 eat the same  〇 have started eating 〇 have started eating  〇 eat more 〇 eat more  〇 eat less 〇 eat less  〇 no longer eat 〇 no longer eat  〇 have never eaten 〇 have never eaten  **13. VEGETABLES 14. PULSES**  **(salads, cooked vegetables, vegetable soups, etc.) (chickpeas, beans, lentils, fava beans, peas, etc.)**  〇 eat the same 〇 eat the same  〇 have started eating 〇 have started eating  〇 eat more 〇 eat more  〇 eat less 〇 eat less  〇 no longer eat 〇 no longer eat  〇 have never eaten 〇 have never eaten |
| --- |

| **EATING HABITS AFTER ONCOLOGICAL THERAPIES**  **(At least six months after surgery)** |
| --- |

| ***MEAT, MEAT PRODUCTS AND MEAT SUBSTITUTES***  *How have your eating habits changed* ***since your diagnosis****?*  *(Only one answer is possible for each question)*  **15. RED MEAT 16. WHITE MEAT**  **(beef, pork, and lamb) (chicken, turkey, and rabbit)**  〇 eat the same 〇 eat the same  〇 have started eating 〇 have started eating  〇 eat more 〇 eat more  〇 eat less 〇 eat less  〇 no longer eat 〇 no longer eat  〇 have never eaten 〇 have never eaten  **17. PROCESSED MEAT 18. PLANT-BASED MEAT SUBSTITUTES**  **(cold meats, salami, cured meats, hot dogs, etc.) (soya burgers, seitan, vegetable nuggets, etc.)**  〇 eat the same 〇 eat the same  〇 have started eating 〇 have started eating  〇 eat more 〇 eat more  〇 eat less 〇 eat less  〇 no longer eat 〇 no longer eat  〇 have never eaten 〇 have never eaten |
| --- |

| ***FISH***  *How have your eating habits changed* ***since your diagnosis****?*  *(Only one answer is possible for each question)*  **19. FISH AND SHELLFISH 20. PRESERVED FISH**  **(fresh, and frozen) (canned and smoked)**  〇 eat the same 〇 eat the same  〇 have started eating 〇 have started eating  〇 eat more 〇 eat more  〇 eat less 〇 eat less  〇 no longer eat 〇 no longer eat  〇 have never eaten 〇 have never eaten |
| --- |

| **EATING HABITS AFTER ONCOLOGICAL THERAPIES**  **(At least six months after surgery)** |
| --- |

| ***MILK AND MILK SUBSTITUTES***  *How have your eating habits changed* ***since your diagnosis****?*  *(Only one answer is possible for each question)*  **21. MILK 22. PLANT-BASED MILK SUBSTITUTES**  **(UHT, fresh, full-cream, semi-/skimmed,** **(soya, rice, oat, kamut, etc.)**  **and lactose-free)**  〇 eat the same 〇 eat the same  〇 have started eating 〇 have started eating  〇 eat more 〇 eat more  〇 eat less 〇 eat less  〇 no longer eat 〇 no longer eat  〇 have never eaten 〇 have never eaten |
| --- |

| ***CHEESE AND EGGS***  *How have your eating habits changed* ***since your diagnosis****?*  *(Only one answer is possible for each question)*  **23. CHEESE 24. EGGS**  **(fresh, mature, grated, spread, low-fat, etc.)**  〇 eat the same 〇 eat the same  〇 have started eating 〇 have started eating  〇 eat more 〇 eat more  〇 eat less 〇 eat less  〇 no longer eat 〇 no longer eat  〇 have never eaten 〇 have never eaten |
| --- |

| ***DRESSINGS AND CONDIMENTS***  *How have your habits changed* ***since your diagnosis****?*  *(Only one answer is possible for each question)*  **25. VEGETABLE OILS 26. ANIMAL FATS**  **(extra-virgin olive oil, and seed oil) (butter, lard, and cream)**  〇 eat the same 〇 eat the same  〇 have started eating 〇 have started eating  〇 eat more 〇 eat more  〇 eat less 〇 eat less  〇 no longer eat 〇 no longer eat  〇 have never eaten 〇 have never eaten |
| --- |

| **EATING HABITS AFTER ONCOLOGICAL THERAPIES**  **(At least six months after surgery)** |
| --- |

| ***DRINKS***  *How have your habits changed* ***since your diagnosis****?*  *(Only one answer is possible for each question)*  **27. ALCOHOLIC DRINKS 28. SOFT DRINKS**  **(wine, beer, spirits, liqueurs, cocktails, etc.) (fruit juice, iced tea, cola, lemonade, tonic water, etc.)**  〇 eat the same 〇 eat the same  〇 have started eating 〇 have started eating  〇 eat more 〇 eat more  〇 eat less 〇 eat less  〇 no longer eat 〇 no longer eat  〇 have never eaten 〇 have never eaten |
| --- |

| ***SWEETS AND DESSERTS***  *How have your eating habits changed* ***since your diagnosis****?*  *(Only one answer is possible for each question)*  **29. HOMEMADE CAKES AND DESSERTS 30. SUGAR SUBSTITUTES**  **(tarts, cakes, pastries, ice cream, desserts, etc.) (agave syrup, barley or rice malt, stevia,**  **other sweeteners, etc.)**  〇 eat the same 〇 eat the same  〇 have started eating 〇 have started eating  〇 eat more 〇 eat more  〇 eat less 〇 eat less  〇 no longer eat 〇 no longer eat  〇 have never eaten 〇 have never eaten |
| --- |

| **FOOD AND NUTRITION: PERSONAL OPINIONS AND SOURCES OF INFORMATION** |
| --- |

| 31. Do you agree that food and nutrition could be linked to cancer? | 〇 no  〇 not much  〇 fairly  〇 very  〇 I don’t know |
| --- | --- |
| 32. After your diagnosis did you ask anyone for information on nutrition? | 〇 YES  〇 NO |
| 32.1. If your answer was YES, who did you go to for this information?  *(****more than one answer is possible****)* | ☐ my own research using literature /Internet/seminars  ☐ general practitioner (GP)  ☐ oncologist  ☐ dietician/nutritionist  ☐ pharmacist  ☐ naturopathic doctor/homeopathic doctor/other non-conventional medical practitioner  ☐ family/friends  ☐ patients with same medical condition |

# Supplementary Tables

**SUPPLEMENTARY TABLE 1**. **Diets started after colorectal cancer diagnosis**. Respondents could select more than one answer. The “other” category includes the following answers: diet, specific diet, eliminating pasta and bread, low-protein diet recommended by a nutritionist, not dieting, and having always been a vegetarian or vegan.

| **Characteristic** | **N = 98***^1^* |
| --- | --- |
| **After diagnosis, did you start a specific diet?: vegetarian diet** | 2/98, 2.0% |
| **After diagnosis, did you start a specific diet?: vegan diet** | 0/98, 0.0% |
| **After diagnosis, did you start a specific diet?: macrobiotic diet** | 0/98, 0.0% |
| **After diagnosis, did you start a specific diet?: high-protein diet** | 1/98, 1.0% |
| **After diagnosis, did you start a specific diet?: blood type diet** | 0/98, 0.0% |
| **After diagnosis, did you start a specific diet?: acid-base/alkaline diet** | 0/98, 0.0% |
| **After diagnosis, did you start a specific diet?: low glycaemic index diet** | 3/98, 3.1% |
| **After diagnosis, did you start a specific diet?: paleo diet** | 0/98, 0.0% |
| **After diagnosis, did you start a specific diet?: raw food diet** | 0/98, 0.0% |
| **After diagnosis, did you start a specific diet?: detox/juice-based diet** | 0/98, 0.0% |
| **After diagnosis, did you start a specific diet?: Kousmine method** | 0/98, 0.0% |
| **After diagnosis, did you start a specific diet?: fasting-mimicking diet** | 0/98, 0.0% |
| **After diagnosis, did you start a specific diet?: other** | 9/98, 9.2% |
| **Reason for following this diet: nutritional deficiencies** | 1/98, 1.0% |
| **Reason for following this diet: support for weight loss** | 4/98, 4.1% |
| **Reason for following this diet: support during chemotherapy/radiotherapy** | 3/98, 3.1% |
| **Reason for following this diet: to fight cancer** | 1/98, 1.0% |
| **Reason for following this diet: other** | 1/98, 1.0% |
| **Who suggested these diets or provided information?: self-directed (books/internet/seminars)** | 1/98, 1.0% |
| **Who suggested these diets or provided information?: general practitioner** | 3/98, 3.1% |
| **Who suggested these diets or provided information?: oncologist** | 1/98, 1.0% |
| **Who suggested these diets or provided information?: dietitian/nutritionist** | 4/98, 4.1% |
| **Who suggested these diets or provided information?: pharmacist** | 0/98, 0.0% |
| **Who suggested these diets or provided information?: naturopath/homeopath/other non-conventional practitioners** | 0/98, 0.0% |
| **Who suggested these diets or provided information?: family members, friends, relatives** | 0/98, 0.0% |
| **Who suggested these diets or provided information?: patients with the same condition** | 0/98, 0.0% |
| **Did you inform your oncologist about following these diets?** | 7/10, 70.0% |
| Missing | 88 |
| *^1^*n/N, % | |
